# Is Atopic Dermatitis Associated with Systemic Metabolic Disturbances? A Systematic Review

**DOI:** 10.3390/ijms26125884

**Published:** 2025-06-19

**Authors:** Mateusz Matwiejuk, Hanna Myśliwiec, Agnieszka Mikłosz, Adrian Chabowski, Iwona Flisiak

**Affiliations:** 1 Department of Dermatology and Venereology, Medical University of Bialystok, 15-089 Białystok, Poland; 2 Department of Physiology, Medical University of Bialystok, 15-089 Bialystok, Poland

**Keywords:** atopic dermatitis, metabolic syndrome, hypertension, hypertriglyceridemia, high-density lipoprotein, obesity, diabetes mellitus, hyperglycemia

## Abstract

Atopic dermatitis (AD) is a chronic, complex, and immunologically mediated skin disease. Its exact cause remains complex, multifaceted and yet to be discovered but is likely related to a combination of immunological, genetic and environmental factors. A medical literature search of PubMed (1992–present), Google Schoolar and Embase was performed using appropriate terms without date limitations in accordance with the Preferred Reporting Items for Systematic reviews and Meta-Analyses (PRISMA) guidelines. Nevertheless, chronic inflammation is believed to be a major player in the development of AD and a causative element in the development of metabolic syndrome (MetS). Metabolic syndrome is a cluster of common metabolic abnormalities including hypertension, insulin resistance, abdominal obesity, reduced high-density lipoprotein (HDL)–cholesterol levels and elevated triglyceride levels. High waist circumference is positively correlated with the risk of atopic dermatitis, but there is no significant correlation between adult-onset atopic dermatitis and hypertension. Some evidence suggests an association between AD and hypertension but only in patients with severe AD. On the other hand, the relationship between AD and hyperglycemia or AD and cholesterol levels seems inconclusive. The aim of this review is to present current knowledge on the association between atopic dermatitis and metabolic syndrome, including each of the components of metabolic syndrome.

## 1. Introduction

### 1.1. Epidemiology of Atopic Dermatitis

Atopic dermatitis (AD) is a chronic skin condition characterized by persistent inflammation, dryness, and itching, affecting mainly children with a prevalence of up to ~20% [1]. The disease develops in 50–60% of patients within the first year of life and in 90% of patients by the age of five [2]. The high incidence of AD and its burden on patients make it the most severe skin disease in the world after acne and psoriasis. The prevalence of AD in European adolescents varies significantly across countries. The highest prevalence rates have been observed in Denmark, Finland, and Hungary, reaching up to 15%. Lithuania has the lowest prevalence rate, with only 1.5% of adolescents affected. The reason for this variation in prevalence is not fully understood, but it is thought to be due to a combination of genetic, environmental, and socioeconomic factors [1]. Similarly, the prevalence of AD in adults varies across countries, ranging from 2.1% to 4.9%. The incidence of AD is generally lower in males compared to females [3]. The balance of hormonal effects on both immunity and the skin barrier likely plays an essential role in regulating the course of AD. First of all, estrogen and progesterone promote Th2/Treg activity and suppress Th1/Th17. Secondly, androgens suppress Th1/Th2/Th17 and induce Treg. Thirdly, dehydroepiandrosterone (DHEA) suppresses Th2 and enhances Th1. The suppressive effects of DHEA and testosterone on Th2 activity are reduced and could lead to an unchecked Th2 response, which is known to play a significant role in the pathogenesis of AD, thereby aggravating symptoms. Sex hormones influence the skin barrier: estrogen fortifies the skin permeability barrier, contrary to progesterone and androgens, which impair the skin permeability barrier [4].

### 1.2. Clinical Presentation of Atopic Dermatitis

AD is a heterogeneous disease with a wide spectrum of lesion morphology, from acute exudation and crusting, subacute lesions to chronic lesions with lichenification and/or prurigo nodules. In addition, clinical symptoms depend on many factors, including the age of disease onset, the severity and extent of cutaneous manifestations, and remissions and recurrences of AD. About 20% of AD cases are classified as mild to severe on various clinical measurement scales [5]. Based on the age of disease onset, AD is categorized into three groups: infants (≤2 years), childhood (2–12 years), and adolescents and adults (13 years and older). In infancy, vesicular lesions, serous exudates, and, in more severe cases, crusts have been described. In early childhood, eczematous lesions develop in flexural areas such as the antecubital fossa, neck, hands, and ankles. Furthermore, these lesions show lichenification rather than exudation. Teenagers and adults have more concentrated eczematous lesions mostly on the head, neck and hands [6]. Importantly, atopic dermatitis develops initially, and then other allergy symptoms can appear, such as food allergy, asthma, and allergic rhinitis (allergic march/atopic triad). Severe clinical symptoms and skin lesions can negatively impact health-related quality of life (QOL) [7]. Recent studies have demonstrated the potential link between atopic dermatitis and metabolic abnormalities. Ali et al. [8], in a systematic review, found a positive association between central obesity and AD, especially in women. However, the association between AD and dyslipidemia or hypertension was ambiguous. The development and course of AD do not appear to be related to hyperglycemia [8].

### 1.3. Pathogenesis of Atopic Dermatitis

**Figure 1:**
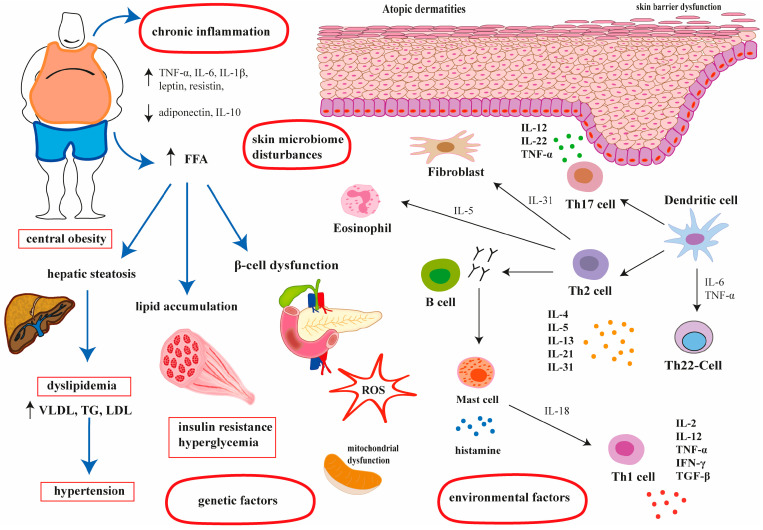
The possible pathogenesis of atopic dermatitis and metabolic syndrome and its relationship. VLDL—very-low-density lipoprotein, TG—triglyceride, LDL—low-density lipoprotein, ROS—reactive oxygen species.

The pathogenesis of AD is multifactorial and includes genetic predisposition, environmental factors (i.e., exposure to airborne or food allergens, pollen, dust mites, the duration of breast-feeding, infections, the use of antibiotics, cosmetics, and detergents), a defect in the epidermal barrier, skin microbiome disturbances, and chronic inflammation (Figure 1) [9]. Several genes have been associated with AD, mainly those encoding structural proteins of the epidermis and key components of the immune system. For example, there is a strong correlation between AD and mutations in the filaggrin gene. The reduced gene expression of filaggrin combined with ceramide deficiency leads to impaired epidermal barrier function (i.e., transepidermal water loss, pH changes, and dehydration). It is estimated that approximately 50% of AD patients have mutations in the filaggrin gene [10]. Other genes involved in epidermal barrier dysfunction and the development of AD include genes encoding epidermal proteases or genes encoding serine protease inhibitors (*SPINK-5/LEKT1*, cystatin A) [11]. Furthermore, activated T helper type 2 (Th2) cells are also responsible for the damage of the epidermal barrier. In addition, the microbial imbalance of skin microbiota significantly contributes to the development and severity of AD. Briefly, atopic dermatitis is characterized by an increased burden of *Staphylococcus aureus* and reduced species diversity compared to healthy and intact skin. A pathogen disrupts the host immune response by directly damaging the skin barrier, interfering with innate immunity, and impairing adaptive immunity. As a consequence, *S. aureus* overwhelms the symbiotic microbiome and may precede and/or trigger disease exacerbations [12]. Eventually, innate and adaptive immune responses play a significant role in the pathogenesis of AD. Although multiple cells reside in the skin, blood-derived immune cells, such as T lymphocytes, plasmacytoid dendritic cells (pDCs), monocytes and granulocytes are crucial players in the development and/or progress of AD skin lesions. The enhanced Th2 inflammatory response and mast cell degranulation triggers the release of cytokines such as interleukins IL-4, IL-5, IL-13, IL-21, and IL31, along with histamine. As a result, eosinophils, basophils, neutrophils and T cells are stimulated, and naïve T cells are differentiated into Th17 and Th1, which exacerbate allergic reactions and are implicated in the development of chronic inflammation, a causal factor of AD. Moreover, in the chronic stage of the disease, IL-18 triggers Th1 cell activation, leading to the release of pro-inflammatory cytokines, i.e., IL-2, IL-12, tumor necrosis factor α (TNF-α), and interferon γ (IFN-γ). These cells contribute to keratinocyte apoptosis and the thickening of the skin [13]. Collectively, the pathogenesis of AD involves the interaction between impaired barrier function, environmental stimuli and dysregulated Th2, Th17, Th-22 immune responses (Figure 1).

### 1.4. Metabolic Syndrome—Definition and Its Association with AD

**Figure 2:**
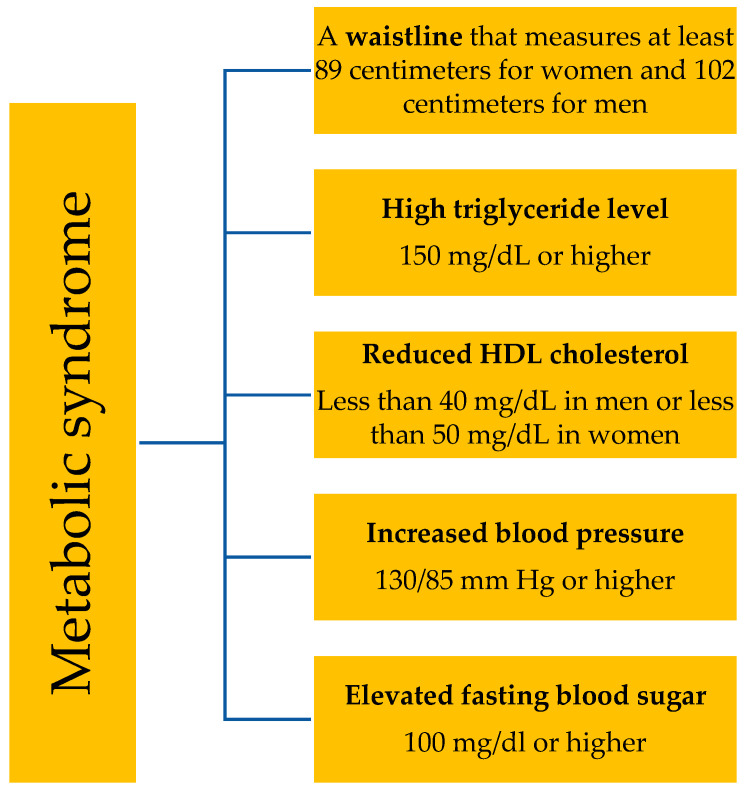
Components of metabolic syndrome.

The National Cholesterol Education Program Adult Treatment Panel III (NCEP ATP III) criteria are widely used to define metabolic syndrome. To diagnose metabolic syndrome, at least three metabolic abnormalities must be present: a waist circumference of over 102 cm for men and 89 cm for women, blood pressure over 130/85 mmHg, a fasting triglyceride level over 150 mg/dL, a fasting HDL cholesterol level less than 40 mg/dL for men and less than 50 mg/dL for women, and a fasting blood glucose level over 100 mg/dL [14]. Figure 2 shows the components of metabolic syndrome. These metabolic disturbances may increase the risk of cardiovascular disease, type 2 diabetes mellitus (T2DM) and stroke. While the exact relationship between AD and MetS is complex and not fully understood, there is growing evidence to suggest that there may be a bidirectional association between the two conditions [15]. Ali et al. [8], in a systematic review, found a positive association between central obesity and AD, especially in women. Central obesity increases pro-inflammatory cytokine concentration such as TNF-α, IFN-γ, IL-1β, IL-2, IL-4, IL-5, IL-6, IL-12, IL-13, IL-17, IL-21, IL-22, and IL-31 and decreases anti-inflammatory cytokines and adipokines such as IL-10 and adiponectin, which leads to immune system imbalances and thus increases the risk of AD (Figure 1). On the contrary, the potential link between AD and dyslipidemia or hypertension is ambiguous. Finally, the development and course of AD do not appear to be related to hyperglycemia [8]. While there is growing research exploring the link between MetS and AD, the association is not yet fully established. Nevertheless, individual components of metabolic syndrome may contribute to the increased incidence of AD and may also exacerbate cutaneous manifestations of the disease.

This review article offers a summary of recent evidence on the association between AD and MetS and between AD and individual components of MetS.

## 2. Materials and Methods

This systematic review was conducted in accordance with the 2020 updated Preferred Reporting Items for Systematic Reviews and Meta-Analyses (PRISMA) guidelines [16,17]. The review protocol was submitted to the Protocols database (registration number 219880).

A medical literature search of PubMed (1992–present), Google Scholar, and Embase, conducted in the autumn and winter of 2024, was performed using appropriate terms without date limitations. The main object of this research was to identify the association between atopic dermatitis and components of metabolic syndrome. Medical subject headline terms included “atopic dermatitis and hypertension”, “atopic dermatitis and cardiovascular diseases”, “atopic dermatitis and high blood pressure”, “atopic dermatitis and obesity”, “atopic dermatitis and dyslipidaemia”, “atopic dermatitis and hypercholesterolemia”, “atopic dermatitis and hyperglycemia”, and “atopic dermatitis and diabetes mellitus”.

Non-English publications, papers with low clinical significance, and duplicated publications were excluded from the analysis. Original and human studies were included in this systematic review. The results of the search strings were combined together, and duplicates were removed. At the initial stage of study selection, the reviewers (M.M. and H.M.) independently examined the titles and abstracts of identified studies to minimize bias in the screening process. Any discrepancies regarding the inclusion or exclusion of studies were discussed between authors and resolved by a third reviewer (A.M.). Finally, the selected eligible articles were fully reviewed.

## 3. Results and Discussion

**Figure 3:**
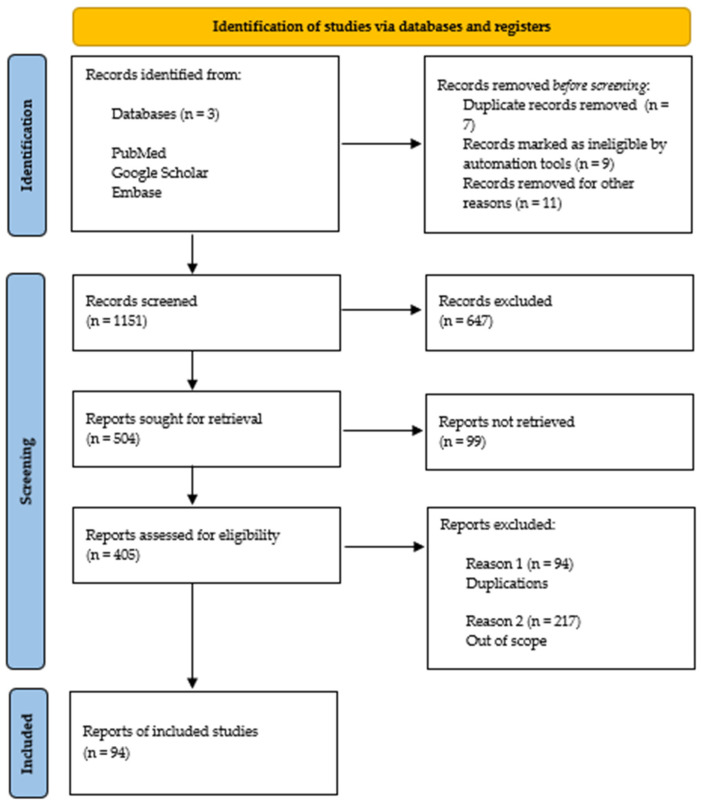
The search process.

According to Figure 3, the search resulted in the screening of 1151 records, of which 405 were assessed for eligibility and 94 were ultimately included in the qualitative synthesis.

### 3.1. The Association Between Metabolic Syndrome and Atopic Dermatitis

Shalom et al. [15] found that AD is associated with a higher prevalence of dyslipidemia and lower prevalence of T2DM compared to the general population. Interestingly, moderate AD and severe AD were associated with a higher prevalence of MetS (17% vs. 9.4% in controls), its components (large waist, elevated fasting blood sugar, hypertension, and dyslipidemia), ischemic heart disease, congestive heart failure, stroke, peripheral vascular disease and cardiovascular morbidity. Importantly, the researchers took into account other factors that could influence metabolic syndrome (age, sex, socioeconomic status, other medical conditions, and smoking) [15]. Collectively, the authors demonstrated the prevalence of various components of metabolic syndrome and the importance of their widespread presence. The prevalence of MetS varies around the world and often corresponds with the prevalence of obesity. There is wide variation in its prevalence based on age, gender, race/ethnicity, and the criteria used for diagnosis (Table 1).

The study by Megna et al. [18] showed a higher prevalence of hypertension (HTN) in patients with adult-onset AD (13.7%) compared to those with persistent AD (2.6%), which begins in childhood and continues into adulthood. Individuals with adult-onset AD experienced a higher frequency of comorbidities compared to those with persistent AD [18] (Table 1).

Shafer et al. [19] noticed that a higher high-density lipoprotein (HDL) cholesterol level corresponds with the prevalence of AD. The authors also found a positive linear association between HDL cholesterol levels and AD. This positive association was also significant for allergic rhinitis and skin prick test (SPT) reactivity. However, for sensitization measured by the RAST, no significant association was observed with HDL levels [19] (Table 1).

Uehara et al. [20] reported that the incidence of HTN was lower in patients with AD compared to the general population. The incidence of disease remained relatively constant in both men and women across the different age groups of AD. Additionally, there was no significant difference in the incidence of HTN between patients with severe and mild AD [20] (Table 1).

The study by Standl et al. [21] showed that patients with AD had only a slightly higher risk of developing hypertension compared to the general population. Moreover, the study also found that AD was not associated with levels of HDL, low-density lipoprotein (LDL), triglycerides (TGs), or total cholesterol (TC) [21] (Table 1).

Drucker et al. [22] found that patients with AD did not have an increased risk of developing HTN and T2DM compared to the general population. In fact, the study revealed an inverse association between AD and these outcomes, meaning that patients suffering from AD were actually at a slightly lower risk of these conditions [22] (Table 1).

Lee et al. [23] reported that MetS and its components, excess body fat around the waist and hypertriglyceridaemia, were positively associated with the presence of AD in women. After adjusting for confounding factors, the odds ratio for female participants with MetS was 2.92, for central obesity, it was 1.73, and for hypertriglyceridaemia, it was 2.20 [23] (Table 1).

In another study, Lee et al. [24] showed that a high waist circumference (≥80 cm) was correlated with an increased risk of developing AD in women, but not in men. This association was statistically significant, with an odds ratio of 2.12 (95% CI 1.11–4.03). Obesity has been hypothesized to disrupt the immune system balance via different mechanisms, for example, changed leptin activity and the increased production of pro-inflammatory factors, like TNF-α, IL-2, IL-4, IL-6, IL-13 and C-reactive protein (CRP). This chronic inflammation and immune dysregulation may contribute to the development and severity of AD. This means that women with a high waist circumference were almost twice as likely to develop AD as women with a normal waist circumference [24] (Table 1).

Lee et al. [25] showed an inverse association between T2DM and AD, while HTN was not linked to AD [25] (Table 1).

Park et al. [26] found no association of impaired fasting glucose (IFG) or T2DM with AD [26] (Table 1).

Silverberg et al. [27] described that AD was linked with a family history of both HTN and T2DM, but not with obesity or hyperlipidemia. These associations were statistically significant, with adjusted odds ratios of 1.88 (95% CI, 1.14–3.10) for HTN and 1.64 (95% CI, 1.02–2.68) for T2DM [27] (Table 1).

Silverberg and Greenland [28] suggested that both high total cholesterol and HTN are associated with an increased risk of developing AD. The odds ratio (OR) for the association of high cholesterol with AD was 1.29 and the OR for the association of hypertension with AD was 1.48 [28] (Table 1).

Radtke et al. [29] showed that obesity is a known risk factor for the development of AD. Obese individuals were 17% more likely to develop AD compared to normal weight subjects. The authors also found that AD was associated with a lower prevalence of hyperlipidemia, HTN, and T2DM [29] (Table 1).

Kwa and Silverberg [30] showed that HTN is a significant risk factor for AD, particularly in women (>50 years of age). The OR for AD associated with HTN was 1.19. It has been concluded that elevated levels of IL-17 and IL-22 in AD patients may contribute to their higher cardiovascular risk, especially hypertension [30] (Table 1).

Augustin et al. [31] noted that hyperlipidemia is linked with AD, whereas HTN is not [31] (Table 1).

A study by Richard et al. [32] showed that the prevalence of metabolic abnormalities like central obesity, dyslipidemia, HTN, and T2DM was higher in patients with psoriasis than in patients with AD. Specifically, the study showed that 62.5% of psoriatic patients met the criteria for metabolic syndrome compared to 34.2% of patients with AD [32] (Table 1).

### 3.2. The Association Between Hypertension and Atopic Dermatitis

Lee et al. [33] reported that adult newly diagnosed with AD patients had a significantly increased risk of developing HTN and other cardiovascular diseases (CVDs) like angina pectoris, myocardial infarction (MI), ischemic stroke and hemorrhagic stroke compared to healthy people [33] (Table 2).

Choi et al. [34] showed that individuals with the atopic triad, which includes asthma, allergic rhinitis, and AD, had a significantly higher prevalence of HTN and atrial fibrillation (AF) compared to the general population. For instance, the adjusted hazard ratio [aHR] for one atopic disease was 1.15, for two atopic diseases, it was 1.34, and similarly, for three atopic diseases, it was 1.35 (*p* < 0.001). However, the mechanisms by which atopic diseases increase the risk of AF are not fully understood. The role of the shift in the Th1/Th2 balance in the pathogenesis of the disease is unknown. On the other hand, cardiac mast cells may cause atrial fibrillation through PDGF-A-mediated fibrosis [34] (Table 2).

Similarly, Rhee et al. [35] found that individuals with multiple allergic diseases, such as asthma, allergic rhinitis, and AD, were at an increased risk of developing HTN compared with individuals with no allergic disease or with a single allergic disease. The prevalence of HTN was 32.7% in the group with multiple allergic diseases compared to 8.1% in the group without allergic diseases [35] (Table 2).

Silverwood et al. [36] found that severe and predominantly active AD were associated with an increased risk of cardiovascular system dysfunction, such as HTN, stroke, MF, and heart failure (HF) [36] (Table 2).

Lundin et al. [37] investigated the association between AD and metabolic risk factors in both men and women. They found that men with AD had a higher body mass index (BMI), percentage of body fat (PBF), systolic blood pressure, total cholesterol, and LDL cholesterol compared to men without AD. No such associations were observed in women [37] (Table 2).

A recent report by Marani et al. [38] that analyzed the prevalence of hypertriglyceridemia and HTN among individuals with AD found that men with AD were more likely to have hypertriglyceridemia and HTN than women with AD. This has been attributed to their different body compositions that may predict different outcomes in women and men. [38] (Table 2).

Kiiski et al. [39] found that people with severe AD were more likely to experience a variety of health problems than those with mild AD. The study followed over 124,000 patients with AD for an average of 7 years. They found that people with severe AD were more likely to develop HTN (OR = 1.15) and atherosclerosis [39] (Table 2).

Artime et al. [40] identified two major comorbidities associated with AD: arterial HTN (36%), and dyslipidemia (35%). These comorbidities can significantly impact the overall health and well-being of AD patients [40] (Table 2).

Yoo et al. [41] reported that individuals with childhood-onset AD (SE = 1,69) had lower rates of T2DM, HTN, and dyslipidemia in comparison to those with adult-onset AD (SE = 2.39) and healthy controls (SE = 0.36) [41] (Table 2).

Arruda et al. [42] showed that hypertension (10.2%) was the main comorbidity in 187 patients with AD, and the most often symptoms were pruritus and erythema. The lesions mainly affected flexural and nonflexural areas, with typical morphology [42] (Table 2).

Wu et al. [43] found that the prevalence of hypertension in the cohort group was approximately 28.8% in 132,460 patients with AD compared to 22.9% in control patients. The study also showed that the association between AD and HTN was stronger in patients with more severe AD [43] (Table 2).

Ivert et al. [44] found that the prevalence of several chronic conditions, including T2DM, hyperlipidemia, and HTN, was higher in patients with severe AD compared to the control group. This difference was also observed in patients with non-severe AD, although to a lesser extent. The study suggested that AD may be a risk factor for these comorbidities, and the early and effective management of AD may help reduce the risk of developing these conditions [44] (Table 2).

Cho et al. [45] showed that the association between AD and HTN was significant only in patients with severe AD. These results suggest that there may be a threshold of AD severity above which the risk of developing HTN increases. Patients with severe atopic dermatitis were classified based on specific treatment criteria: at least three prescriptions for systemic immunosuppressants (e.g., methotrexate, cyclosporine, azathioprine, or mycophenolate mofetil) or systemic corticosteroids or at least three superpotent topical corticosteroids (e.g., clobetasol) combined with at least three cycles of phototherapy. In 2010, the distribution of AD cases by severity was as follows: severe AD: 7.43% of all cases and moderate AD: 19.26% of all cases [45] (Table 2).

Similarly to the above-mentioned study, the study by Kok et al. [46] showed that an increased BMI (overweight or obesity), as well as HTN, hyperlipidaemia, and T2DM, was significantly correlated with the occurrence of moderate-to-severe AD. Overweight individuals (BMI ≥ 25 kg/m^2^) were 23% more likely to have moderate-to-severe AD (OR 1.23 (95% CI: 1.12–1.35, *p* < 0.001)). In turn, obese patients (BMI ≥ 30.0 kg/m^2^) were 44% more likely to have moderate-to-severe AD, OR: 1.44 (95% CI: 1.28–1.63, *p* < 0.001). In individuals with one or more metabolic conditions like HTN, T2DM or hyperlipidemia, an even stronger association with moderate-to-severe AD was found (the OR ranged from 1.43 to 6.85, *p* < 0.001) [46]. In contrast to the above-mentioned studies, Lee et al. [25] revealed no significant correlation between HTN and adult-onset AD [25] (Table 2).

In summary, a positive association between vascular inflammation and AD is observed. Specifically, a strong link was found between vascular inflammation and the presence of Th2-related products. These products include molecules like CCL17 and CCL22, which are known to be involved in the Th2 immune response. In essence, the study suggests that even in young AD patients without overt CVD, there is a measurable level of vascular inflammation that correlates with markers of the Th2 immune response, which is a hallmark of AD. This finding could imply a systemic component to AD, potentially linking skin inflammation to an increased risk of cardiovascular disease [33] (Table 2).

### 3.3. The Association Between Central Obesity and Atopic Dermatitis

Jung et al. [47] studied the influence of weight loss in adults on the severity of AD. Patients in the weight loss group had a significant decrease in BMI from 29.37 to 24.42 at week 12 (*p* = 0.043) and decreased AD symptoms. Additionally, a positive correlation between BMI and eczema area and severity index (EASI) scores (comprising erythema, scaling, infiltration, and itching) was found [47] (Table 3).

Silverberg et al. [48] revealed a significant association between obesity in children and an increased risk of developing AD. Using conditional logistic regression, the study found that obese children are twice as likely to experience atopic dermatitis compared to their normal-weight counterparts. Moreover, the study established a link between obesity and a more severe presentation of atopic dermatitis, as measured by the EASI (erythema, scaling, infiltration, and itching) score [48] (Table 3).

Similar results were presented by Kusunoki et al. [49]. The authors highlighted that childhood obesity is positively associated with both the prevalence and severity of AD. They found that obese children with AD were more likely to have severe symptoms than obese children without AD or non-obese children with AD. This suggests that obesity may worsen the course of AD in children. The authors speculate that a severe course of AD in obese children may be related to the increased leptin content in obese individuals. Leptin affects the immune system by increasing the Th1 response and increasing the synthesis of pro-inflammatory cytokines like IFN-γ and TNF-α, which are usually involved in the development of allergic reactions [49] (Table 3).

Luo et al. [50] found that obesity was significantly associated with AD in Chinese adults. The authors found that obese adults were 2.7 times more likely to have AD than their non-obese counterparts [50] (Table 3).

Koutroulis et al. [51] noticed that increased BMI was associated with increased severity of AD in children older than two years, but not in children younger than two years. The researchers emphasized that children with a BMI above the 85th percentile were more likely to have severe AD than children with a BMI below the 15th percentile. Although elevated levels of pro-inflammatory markers such as TNF-α, IL-6, and C-reactive protein are found in children with AD, their levels are unchanged in obese and normal-weight children with AD, suggesting that a different mechanism likely accounts for the association between obesity and AD severity. Importantly, in children under two years of age, increased body mass for length does not correlate with AD severity. It is speculated that AD severity may be influenced more by genetic issues like filaggrin mutations or behavioral factors, such as diet or allergen exposure, than obesity-related inflammation. Moreover, the inflammatory response to obesity in infants may differ to that in older children, possibly due to their immature immune system [51] (Table 3).

Kilpelainen et al. [52] discovered that the risk of AD increased linearly with BMI among women but not men. The researchers analyzed data from 10,667 Finnish first-year university students aged 18–25 years. They noticed that women with a BMI of 20.0–22.4 were 21% more likely to have AD than women with a BMI below 20.0. Women with a BMI of 25.0–27.4 were 41% more likely to have AD than women with a BMI below 20.0. This association was not observed in men. The authors presumed that AD in adults is more heterogeneous than allergic rhinoconjunctivitis with respect to IgE-mediated sensitization; therefore, a linear association between BMI and AD was not expected [52] (Table 3).

Silverberg et al. [53] showed that obesity in adults was associated with an increased risk of AD, but not non-atopic dermatitis. Precisely, it was presented that obese adults were 43% more likely to have AD than normal-weight adults, but there was no increased risk of NAD among obese adults [53] (Table 3).

Xie et al. [54] reported that individuals with AD for more than one year had a higher BMI than healthy controls. Additionally, the prevalence of obesity was significantly higher in individuals with AD for more than one year compared to both newly diagnosed AD cases and healthy controls [54] (Table 3).

Drucker et al. [55] reported that maternal gestational weight gain (GWG) was associated with an increased risk of AD in offspring. The researchers found that the children of mothers who gained more than 16 kg during pregnancy were more likely to develop AD than the children of mothers who gained less weight. The association was strongest for mothers with a pre-pregnancy BMI greater than 25. The authors explain this association by two main mechanisms: inflammation and host-microbiome interactions. Excessive GWG is associated with a higher CRP level that, during pregnancy, can create a pro-inflammatory environment, influencing fetal immune development. In addition, the gut microbiome undergoes significant changes during the period of pregnancy, which are influenced by metabolic changes [55] (Table 3).

Zhang et al. [56] revealed that class I obesity (BMI 30–34.9 kg/m^2^) was positively associated with moderate-to-severe AD in the Dutch general population [56] (Table 3).

Ascott et al. [57] found that people with AD had a slightly higher risk of being overweight or obese compared to people without AD (OR = 1.08). It was revealed that in patients suffering from severe eczema, there was no link found between the skin condition and being overweight or obese [57] (Table 3).

Vehapoglu et al. [58] investigated the association between obesity and atopic allergic disease (AAD), for instance, AD in pre-pubertal children. The study found that both obese (OR= 1.71; 95% CI: 1.08–2.71, *p* = 0.021) and overweight (OR = 1.62; 95% CI: 1.06–2.50, *p* = 0.026) children were at an increased risk of developing AAD compared to normal-weighted children. This association was observed regardless of gender. On the other hand, there was no significant association between underweight status and AAD (OR = 1.03; 95% CI: 0.63–1.68, *p* = 0.894) [58] (Table 3).

Kim et al. [59] found that obesity and overweight are associated with an increased risk of allergic diseases, including AD. This association was statistically significant, with an OR of 1.11 for AD [59] (Table 3).

Lin et al. [60] found that adolescents with both restricted fetal growth and a high BMI had a significantly increased risk of developing AD. The odds ratio of developing AD was 1.64 [60] (Table 3).

In contrary to the above-mentioned studies, Harpsøe et al. [61] did not find an association between maternal obesity or gestational weight gain and the risk of atopic eczema or hay fever. The study followed a cohort of 38,874 mother–child pairs from the Danish National Birth Cohort from 1996 to 2002 [61] (Table 3).

Similarly, Sybilski et al. [62] found no significant association between BMI and the prevalence of AD regardless of age, gender or living conditions. The study included a total of 33,184 participants from Poland [62] (Table 3).

Saadeh et al. [63] found that a high BMI was not significantly (*p* < 0.05) associated with atopic eczema in either of the groups (normal weight and high BMI) of 6733 randomly selected French schoolchildren aged 9–11 years [63] (Table 3).

A study by von Kries et al. [64] found that hay fever and atopic eczema were unrelated to weight in 9357 of 6-year-old German children [64] (Table 3).

Yoo et al. [65] did not find a significant association between overweight status and AD in either gender of 717 adolescents from Seoul, Korea [65] (Table 3).

Similarly, Vlaski et al. [66] reported no significant association between overweight and atopic eczema in Macedonian adolescents [66] (Table 3).

Kajbaf et al. [67] revealed no significant association between obesity and atopic eczema among 903 school-aged children (7 to 11 years of age). The researchers assessed the participants’ BMI and current eczema using the SCORAD (SCORing Atopic Dermatitis) index [67] (Table 3).

Nicholas et al. [68] found that AD was associated with lower height and a higher BMI in children but that these associations were not statistically significant as children aged. The study followed children with and without AD for 14 years and found that the differences in height and BMI between the two groups decreased over time. At 5.5 years of age, the difference in BMI between the two groups was no longer statistically significant [68] (Table 3).

Immune imbalance through the increased production of leptin and pro-inflammatory factors such as TNF-α, IL-2, IL-4, IL-6, IL-13, and CRP is a potential mechanism linking central obesity to AD. This chronic inflammation and immune dysregulation may positively contribute to the development and severity of AD [24] (Table 1).

### 3.4. The Association Between Hypertriglyceridaemia and Atopic Dermatitis

Kim et al. [69] noticed that children with AD had significantly higher levels of TC and TG compared to children without AD. Additionally, the severity of AD, as measured by the SCORAD index, was positively associated with TC and TG levels and negatively associated with HDL levels. These findings suggest that there may be an underlying connection between AD and lipid metabolism; for instance, cholesterol may be linked to the chronic course of AD [69] (Table 4).

Similarly, Baek et al. [70] investigated the association between AD and lipid profiles in children. They found that children with AD had significantly higher levels of TC and TG compared to children without AD. Additionally, the SCORAD index, which measures the severity of AD, was positively associated with TC and TG levels [70] (Table 4).

Agón-Banzo et al. [71] stated that patients with AD had significantly higher levels of TG compared to healthy controls. Additionally, the severity of AD was positively associated with TG levels [71] (Table 4).

Seong et al. [72] investigated the association between AD and dyslipidemia in adolescents. They found that patients with AD had significantly higher levels of TG compared to healthy controls [72] (Table 4).

Li et al. [73] investigated the role of triglycerides in cutaneous antimicrobial defense in patients with AD and *S. aureus* colonization. They found that patients with AD and *S. aureus* colonization had significantly lower levels of certain triglycerides, including TG46:1, TG48:1, TG48:2, TG50:1, TG50:2, and TG50:3. Additionally, they revealed that a decreased level of TG46:2 was associated with increased basal transepidermal water loss (TEWL), a measure of skin barrier disruption. These findings suggest that triglycerides may play a role in cutaneous antimicrobial defense by maintaining the skin barrier function and preventing the entry of *Staphylococcus aureus* into the skin [73] (Table 4).

Elevated triglycerides (TGs) are strongly linked to increased inflammation and play a significant role in the development and progression of cardiovascular diseases, particularly atherosclerosis. Pro-inflammatory signaling, particularly involving cytokines like TNF-α and IL-6, is implicated in both AD and hypertriglyceridemia [69] (Table 4).

### 3.5. The Association Between HDL Level and Atopic Dermatitis

Schafer et al. [19] investigated the association between HDL levels and AD. They found that HDL was positively associated with AD in men [19] (Table 5).

Trieb et al. [74] observed a significant depletion in the atopic dermatitis–HDL content of apolipoprotein C-III, apolipoprotein E, cholesteryl ester, free cholesterol, lyso-phosphatidylcholine (especially 16:0 species), and phosphatidylethanolamine compared to the control subjects. In addition, a significant enrichment of atopic dermatitis–HDL in apolipoprotein A-II, the acute-phase protein like serum amyloid A, and phosphatidylinositol was noticed. However, median C-reactive protein levels of AD patients were unchanged [74] (Table 5).

A study by Lundin et al. [37] shows that young adolescents with AD had lower HDL levels compared to those without AD. Surprisingly, this lower HDL level was associated with a reduced risk of CVD in the AD group [37] (Table 5).

Kusunoki et al. [75] found that patients with AD had significantly lower HDL levels than healthy controls. On the other side, no association between HDL-C levels and IgE levels in AD patients was spotted [75] (Table 5).

In contrast to the above-mentioned studies, Leigh et al. [76] stated that there was no significant difference in HDL-C levels between adolescents with AD and those without AD [76] (Table 5).

It seems that paraoxonase, an enzyme well known for its antioxidant and anti-inflammatory properties, is primarily associated with a low concentration of HDL [74] and plays a role in the pathogenesis of low-grade inflammation in AD. In turn, the presence of chronic low-grade inflammation is associated with an increased risk of AD (Table 5).

### 3.6. The Association Between *Hyperglyceamia and* Atopic Dermatitis

Lee et al. [77] found that adults with AD have a significantly increased risk of developing T2DM compared to those without AD. The adjusted hazard ratio (aHR) was 1.44. This association was observed even after adjusting for other risk factors for T2DM, such as age, sex, and BMI [77] (Table 6).

A study by Hung et al. [78] revealed that children whose parent has an autoimmune disease, such as T1DM, have an increased risk of developing AD. The adjusted odds ratio OR was 2.071 [78] (Table 6).

Lu et al. [79] provided evidence of a common genetic basis between AD and T1DM and T2DM. The ORs for T1D and T2D were 1.19 and 1.07, respectively [79] (Table 6).

Woo et al. [80] showed that in patients with type 2 diabetes, the incidence of dementia was significantly higher in those with AD than in those without this disease (HR = 1.158). Additionally, in patients with T2DM, the risks of incident Alzheimer’s disease were greater in those with AD than in those without AD (HR= 1.159) [80] (Table 6).

Olesen et al. [81] conducted a retrospective case–control study to investigate the association between AD and T1DM in children. They found that the collective incidence of AD up to age 15 years with T1DM was about two-thirds of that among nondiabetic controls [81] (Table 6).

In the contrary, Li et al. [82] found that the overall incidence rate of AD was significantly higher in the T1DM cohort compared to the non-T1DM cohort, even after adjusting for potential risk factors [82] (Table 6).

Kumar et al. [83] showed that GDM was significantly associated with AD in term deliveries. Infants born to mothers with GDM were 7.2 times more likely to develop AD than infants born to mothers without GDM. The authors speculated that there is a potential link between GDM in mothers and impaired immune development in their infants, mediated by changes in adipokine levels. Mothers struggling with GDM tend to have higher levels of TNF-alpha, leptin, and visfatin and lower levels of adiponectin. The study also showed that the association between GDM and AD was stronger in the case of food allergies than in the case of allergies to inhaled anesthetics [83] (Table 6).

Karavanaki et al. [84] found that atopy, including atopic eczema, was more common in children from higher socioeconomic classes with T1DM. There are several potential explanations for this association. First, children from higher socioeconomic classes tend to have a more refined diet, which may be less rich in certain nutrients that are important for immune function. Second, children from higher socioeconomic classes tend to live in more indoor environments, which may be more conducive to the development of allergens. Third, the hygiene hypothesis suggests that exposure to certain germs in early childhood may help to protect against allergies later in life. Children from higher socioeconomic classes may have less exposure to these germs [84] (Table 6).

Li et al. [85] found that there was no significant association between AD and type 1 diabetes mellitus (T1DM) in the first year after AD diagnosis, and there was no increased risk of T1DM in AD patients thereafter [85] (Table 6).

Similarly, Berg et al. [86], in a large nationwide Danish case–cohort study, did not find a significant association between T1DM and AD, even when considering different severity levels of AD or whether the diagnosis was made by a dermatologist or not. However, the study showed that children with AD who were prescribed steroid lotion had an increased risk of developing T1DM [86] (Table 6).

Stromberg et al. [87] noticed that the prevalence of atopic eczema was similar in children with T1DM and healthy controls. Moreover, the two groups also did not differ significantly in terms of clinical findings, skin prick test results, total serum IgE levels, or the presence of circulating IgE antibodies to allergens [87] (Table 6).

Campanati et al. [88] proved that there was no significant difference in the prevalence of T2DM between patients with different severities of AD in contrast to healthy controls [88] (Table 6).

Rosenbauer et al. [89] found that the risk of developing T1DM was significantly lower among children with AD compared to children without AD (adjusted HR = 0.82, 95% CI: 0.75–0.89). This suggests that the occurrence of AD may protect against the development of T1DM [89] (Table 6).

Stene et al. [90] found that AD was associated with a lower risk of T1DM, even after adjusting for various potential confounding factors. This suggests that AD may indeed confer some protection against the development of T1DM [90] (Table 6).

Thomsen et al. [91] found that in twins, the diabetic twin had a significantly lower risk of AD compared to the non-diabetic twin. This suggests that there may be genetic factors contributing to both conditions, but these factors do not completely overlap. The negative correlation between genetic factors for T1DM and AD (r = −0.30) further supports this hypothesis [91] (Table 6).

Cakir et al. [92] reported that the prevalence of atopy, asthma, and atopic eczema was lower in children with T1DM compared to the control group. This suggests that there may be a weak association between T1DM and the aforementioned conditions [92].

Meerwaldt et al. [93] also showed that children with T1DM were less likely to suffer from atopic eczema (OR = 0.693) than the control group [93] (Table 6).

Kim et al. [94] noticed that the prevalence of HTN, T2DM, and obesity was lower in subjects with AD compared to those without AD in a Korean population [94] (Table 6).

The chronic, low-grade systemic inflammation and specific cytokines (particularly Th2-driven components Il-4, Il-5, and Il-13, with additional input from IL-17) that characterize AD could positively contribute to the development of insulin resistance and ultimately T2DM. The similar inflammatory profiles observed in both conditions provide strong correlational evidence for an inflammatory link [79] (Table 6).

## 4. Limitations

Scientific knowledge about the etiology of AD has advanced significantly in recent years. The complexity of AD pathogenesis is responsible for the enormous variability in AD phenotypic expression, intensity, and clinical course, which makes it difficult to compare the results obtained in clinical studies. In addition, there are many studies that differ significantly in terms of experimental design like animal models or small–scale clinical trials, which also affects the comparability and reproducibility of results.

## 5. Conclusions

Atopic dermatitis is frequently accompanied by metabolic syndrome or its individual components. In the present study, the current state of knowledge on the potential association between the atopic dermatitis and each component of metabolic syndrome was shown. There was a significant positive association between central obesity (measured by waist circumference) and AD. On the other hand, the association between hyperglyceamia, hypertiglyceridemia or cholesterol levels and atopic dermatitis was inconclusive. Nevertheless, some available data provide contradictory results and neglect the association between atopic dermatitis and individual components of metabolic syndrome. While the exact mechanisms linking these conditions are complex, chronic inflammation is a shared pathway. Therefore, further studies are needed to fully elucidate the mechanisms involved in their development, possible clinical implications, and benefits.

## Figures and Tables

**Table 1:** A summary of the studies on metabolic syndrome in atopic dermatitis.

Author	Year	Population	Key Observation
Metabolic Syndrome in Atopic Dermatitis			
Shalom et al. [15]	2016	N0—116,812 healthy people N1—116,816 patients with AD	Moderate and severe AD were associated with a higher prevalence of MetS.
Megna et al. [18]	2017	151 patients with persistent AD 102 patients with adult-onset AD	A higher prevalence of HTN in patients with adult-onset AD compared to those with persistent AD was found.
Schafer et al. [19]	2003	N0—1491 healthy people N1—46 patients with AD	HDL-cholesterol level was associated with a higher prevalence of AD.
Uehara et al. [20]	2002	521 patients with active AD 87 adults with “healed” AD	The incidence of HTN was lower in patients with AD compared to the general population.
Standl et al. [21]	2017	N0—30,047 healthy people N1—10,788 patients with AD	In patients with AD, the risk of developing hypertension was only slightly higher compared to the general population.
Drucker et al. [22]	2017	N0—237,740 healthy patients N1—21,379 patients with AD	AD patients were not found to be at increased risk of developing HTN or T2DM compared to the general population.
Lee et al. [23]	2017	5007 subjects (2142 men and 2865 women)	MetS and its individual components like central obesity and hypertriglyceridaemia were positively associated with the presence of AD in women.
Lee et al. [24]	2016	5202 Korean adults aged 19–40 years	High waist circumference (≥80 cm) was associated with an increased risk of AD in women.
Lee et al. [25]	2014	47,351 participants	An inverse association has been demonstrated between T2DM and AD.
Park et al. [26]	2017	2342 subjects	There was no association between IFG and AD nor between T2DM and AD.
Silverberg et al. [27]	2015	N0—143 healthy controls N1—132 children with AD	AD was linked with a family history of both HTN and T2DM, but not with obesity or hyperlipidemia.
Silverberg et al. [28]	2015	34,525 participants	High cholesterol and HTN were associated with an increased risk of developing AD.
Radtke et al. [29]	2013	37,456 patients with psoriasis, 48,140 patients with AD	Obese individuals were 17% more likely to develop AD compared to those with normal weight.
Kwa et al. [30]	2017	164,868 patients with AD	HTN was a significant risk factor for AD, particularly in women.
Augustin et al. [31]	2015	30,354 patients with AD	Hyperlipidemia was linked with AD while HTN is not associated with AD.
Richard et al. [32]	2019	931 patients with AD, 190,891 patients with psoriasis	Obesity, dyslipidemia, HTN, and T2DM were more prevalent among patients with psoriasis than patients with AD.

Abbreviations: N0—control group, N1—study group, HDL—high-density lipoprotein, IFG—impaired fasting glucose.

**Table 2:** A summary of the studies on high blood pressure in atopic dermatitis.

Author	Year	Population	Key Observation
High Blood Pressure in Atopic Dermatitis			
Lee et al. [33]	2023	N0–40,512 control subjects without AD N1—40,512 individuals with AD	Patients with AD had a significantly increased risk of developing HTN.
Choi et al. [34]	2021	N0—136,253 subjects with AF N1—30,300 patients with AD in the atopic group N2—105,953 patients with AD in the nonatopic group	Individuals with the atopic triad, which includes asthma, allergic rhinitis, and AD, had a significantly higher prevalence of HTN and AF.
Rhee et al. [35]	2020	9,548,939 individuals older than 20 years	Patients suffering from asthma, allergic rhinitis, and AD were at increased risk of developing HTN.
Silverwood et al. [36]	2018	N0—1,528,477 patients without atopic eczema; N1—387,439 patients with atopic eczema	AD was associated with an increased risk of cardiovascular outcomes, such as HTN.
Lundin et al. [37]	2023	N0—1850 patients without AD; N1—420 patients with AD	Men with AD had higher SP levels compared to men without AD.
Marani et al. [38]	2023	686 adult patients with AD (357 males and 329 females)	Men with AD were significantly more likely to have hypertriglyceridemia and HTN compared to women with AD.
Kiiski et al. [39]	2023	124,038 patients with AD	People with severe AD were more likely to develop HTN (OR = 1.15) and atherosclerosis than those with mild AD.
Artime et al. [40]	2022	1995 patients with AD	Two major comorbidities were associated with AD: arterial HTN (36%), and dyslipidemia (35%).
Yoo et al. [41]	2022	N0—32,086 healthy people N1—383 child-onset AD N2—440 adult-onset AD	Childhood-onset AD (SE = 1.69) had lower rates of comorbid T2DM, HTN, and dyslipidemia in comparison to those with adult-onset AD (SE = 2.39).
Arruda et al. [42]	2023	N0—1995 patients N1—187 patients with AD	The main comorbidity of the 187 patients with AD was HTN (10.2%).
Wu et al. [43]	2021	N0—397,380 healthy patients N1—132,460 patients with AD	The association between AD and HTN was stronger in patients with more severe AD.
Ivert et al. [44]	2019	N0—1,022,435 healthy patients N1—104,832 patients with AD	The incidence of HTN was higher in patients with severe AD compared to controls.
Cho et al. [45]	2020	12,780 patients with AD	The association between AD and HTN was only significant in patients with severe AD.
Kok et al. [46]	2019	10,077 patients with AD	HTN was significantly correlated with having moderate-to-severe AD.
Lee et al. [25]	2014	47,351 participants	No significant correlation between HTN and adult AD.

Abbreviations: HTN—hypertension, AF—atrial fibrillation, SP—systolic blood pressure.

**Table 3:** A summary of the studies on obesity in atopic dermatitis.

Author	Year	Population	Key Observation
Obesity in Atopic Dermatitis			
Jung et al. [47]	2020	N1—20 normal-weight patients with AD N2—obese patients with AD	Weight reduction was associated with significant improvement in AD symptoms.
Silverberg et al. [48]	2011	N0—778 healthy patients N1—389 patients with AD	There was a significant association between obesity in children and an increased risk of developing AD.
Kusunoki et al. [49]	2008	50,086 questionnaires	A large waist was positively associated with both the prevalence and severity of AD.
Luo et al. [50]	2013	N0—532 healthy patients N1—266 atopic patients	Obesity and overweight were associated with an increased risk of allergic diseases, including AD.
Koutroulis et al. [51]	2015	104 patients with AD	An increased BMI was associated with increased severity of AD in children older than two years.
Kilpelainen et al. [52]	2016	10,667 students	AD increased linearly with BMI among women but not men
Silverberg et al. [53]	2011	2090 participants	Obesity in adults was associated with an increased risk of AD.
Xie et al. [54]	2017	N0—2217 control patients N1—3515 atopic patients	Patients suffering from AD for more than one year had a higher BMI than healthy controls.
Drucker et al. [55]	2019	N0—11,211 control patients N1—2058 patients with AD	Maternal gestational weight gain was associated with an increased risk of AD in offspring.
Zhang et al. [56]	2022	56,896 participants	Class I obesity (BMI 30–34.9 kg/m^2^) was positively associated with moderate-to-severe AD.
Ascott et al. [57]	2020	N0—849,722 healthy patients N1—1,441,746 patients with AD	People with AD had a slightly higher risk of being overweight or obese compared to people without AD.
Vehapoglu et al. [58]	2021	N0—429 healthy patients N1—278 atopic patients	Obese and overweight children were at an increased risk of developing AAD, including AD in comparison to children with normal weight.
Kim et al. [59]	2018	703,869 participants (363,180 boys and 340,689 girls)	Obesity and overweight were associated with an increased risk of allergic diseases, including AD.
Lin et al. [60]	2015	N0—69,012 control patients N1—5676 patients with AD	Adolescents with both restricted fetal growth and a high BMI had a significantly increased risk of developing AD.
Harpsøe et al. [61]	2012	38,874 children and their mothers	There was no association between maternal obesity or gestational weight gain and the risk of atopic eczema or hay fever.
Sybilski et al. [62]	2013	4783 participants	There was not a significant association between BMI and the prevalence of AD in children and adults.
Saadeh et al. [63]	2014	6733 schoolchildren	A high BMI was not significantly (*p* < 0.05) associated with atopic eczema.
Kries et al. [64]	2001	9357 children	Atopic eczema was unrelated to children’s weight.
Yoo et al. [65]	2009	717 adolescents	There was not a significant association between overweight and AD.
Vlaski et al. [66]	2006	2926 young adolescents	There was no significant association between being overweight and atopic eczema.
Kajbaf et al. [67]	2011	903 children	There was no significant association between obesity and atopic eczema.
Nicholas et al. [68]	2022	N0—8777 healthy patients N1—1834 atopic patients	AD was associated with lower height and a higher BMI in children.

Abbreviations: AAD—atopic allergic disease.

**Table 4:** A summary of the studies on hypertriglyceridemia in atopic dermatitis.

Author	Year	Population	Key Observation
*Hypertriglyceridemia* in Atopic Dermatitis			
Kim et al. [69]	2021	Subset I—248 patients Subset II—52,725 patients	Children with AD had significantly higher levels of TC and TG compared to children without AD.
Baek et al. [70]	2020	N0—179 controls N1—69 atopic patients	Patients with AD had significantly higher levels of TC and TG compared to children without AD.
Agón-Banzo et al. [71]	2019	N0—105 healthy controls N1—134 children diagnosed with AD	Patients with AD had significantly higher levels of TG compared to healthy controls.
Seong et al. [72]	2023	1617 Korean adolescents	Patients with AD had significantly higher levels of TG compared to healthy controls.
Li et al. [73]	2017	N0—15 healthy individuals N1—27 patients with AD	Patients with AD and *S. aureus* colonization had significantly lower levels of TG46:1, TG48:1, TG48:2, TG50:1, TG50:2, and TG50:3.

**Table 5:** A summary of the studies on HDL in atopic dermatitis.

Author	Year	Population	Key Observation
HDL in Atopic Dermatitis			
Schafer et al. [19]	2003	N0—1491 controls N1—46 patients with AD	The level of HDL was positively associated with AD in men.
Trieb et al. [74]	2019	N0—19 controls N1—20 patients with AD	HDL may play a role in the pathogenesis of low-grade inflammation in AD.
Lundin et al. [37]	2023	N0—1850 patients without AD N1—420 patients with AD	Young adolescents with AD had lower HDL levels compared to those without AD.
Kusunoki et al. [75]	2011	654 children	Patients with AD had significantly lower HDL levels than healthy controls.
Leigh et al. [76]	2021	N0—1782 healthy controls N1—230 patients with AD	No significant difference in HDL-C levels between adolescents with AD and those without AD was noticed.

Abbreviations: AD—atopic dermatitis, HDL—high-density lipoprotein, HDL-C—high-density lipoprotein cholesterol.

**Table 6:** A summary of the studies on elevated blood sugar levels in atopic dermatitis.

Author	Year	Population	Key Observation
Elevated Blood Sugar Levels in Atopic Dermatitis			
Lee et al. [77]	2023	36,692 individuals with AD	Adults with AD have a significantly increased risk of developing T2DM compared to adults without AD.
Hung et al. [78]	2022	N0—862,612 children without AD N1—312,329 children with AD	Children who have a parent with an autoimmune disease, such as T1DM, are at an increased risk of developing AD.
Lu et al. [79]	2023	N0—95,464 individuals without AD N1—21,399 individuals with AD	There is a shared genetic basis between AD and T1DM and T2DM.
Woo et al. [80]	2023	N0—2,643,602 patients without AD N1—38,391 patients with AD	Patients suffering from T2DM were characterized by a significantly elevated incidence of dementia in those with AD compared to those without AD.
Olesen et al. [81]	2001	N0—7683 patients without diabetes mellitus N1—817 patients with diabetes mellitus	An incidence of AD up to age 15 years with T1DM was about two-thirds of that among nondiabetic controls.
Lu et al. [82]	2015	N0—12,725 patients without T1DM N1—3386 patients with T1DM	The overall incidence rate of AD was significantly higher in patients with T1DM.
Kumar et al. [83]	2009	N0—680 total amount of children N1—231 children with AD	GDM was significantly associated with AD in term deliveries.
Karavanaki et al. [84]	2008	N0—150 control patients N1—127 patients with T1DM	Atopic eczema was more common in higher socioeconomic classes children suffering from T1DM.
Li et al. [85]	2023	N0—1,585,844 controls N1—396,461 patients with AD	There was no significant association between AD and T1DM.
Berg et al. [86]	2023	N0—45,656 controls N1—4111 patients with T1DM	No significant association between T1DM and AD was noted.
Stromberg et al. [87]	1995	N0—78 healthy patients N1—61 children with T1DM	The prevalence of atopic eczema was similar in children with T1DM and healthy controls.
Campanati et al. [88]	2021	684 participants	There was no significant difference in the prevalence of T2DM between patients with different severities of AD.
Rosenbauer et al. [89]	2003	N0—1871 patients without T1DM N1—760 patients with T1DM	T1DM was significantly lower among children with AD compared to children without AD.
Stene et al. [90]	2004	N0—1668 controls N1—554 patients with T1DM	AD was associated with a lower risk of T1DM.
Thomsen et al. [91]	2011	N1—5289 patients with AD N2—143 patients with T1DM	The diabetic twin had a significantly lower risk of AD compared to the non-diabetic twin.
Cakir et al. [92]	2008	N0—100 healthy controls N1—52 children with T1DM	The prevalence of atopy, asthma, and atopic eczema was lower in children with T1DM compared to the control group.
Meerwaldt et al. [93]	2002	N0—777 control patients N1—188 patients with T1DM	Children with T1DM suffered less from atopic eczema than the control group.
Kim et al. [94]	2015	18,066 participants	The prevalence of HTN, T2DM, and obesity was lower in subjects with AD.

Abbreviations: T1DM—type 1 diabetes mellitus.

## Data Availability

Not applicable.
